# A 3D-printed hand-powered centrifuge for molecular biology

**DOI:** 10.1371/journal.pbio.3000251

**Published:** 2019-05-21

**Authors:** Gaurav Byagathvalli, Aaron Pomerantz, Soham Sinha, Janet Standeven, M. Saad Bhamla

**Affiliations:** 1 Lambert High School, Suwanee, Georgia, United States of America; 2 School of Chemical & Biomolecular Engineering, Georgia Institute of Technology, Atlanta, Georgia, United States of America; 3 Department of Integrative Biology, University of California, Berkeley, California, United States of America

## Abstract

The centrifuge is an essential tool for many aspects of research and medical diagnostics. However, conventional centrifuges are often inaccessible outside of standard laboratory settings, such as remote field sites, because they require a constant external power source and can be prohibitively costly in resource-limited settings and Science, technology, engineering, and mathematics (STEM)-focused programs. Here we present the 3D-Fuge, a 3D-printed hand-powered centrifuge, as a novel alternative to standard benchtop centrifuges. Based on the design principles of a paper-based centrifuge, this 3D-printed instrument increases the volume capacity to 2 mL and can reach hand-powered centrifugation speeds up to 6,000 rpm. The 3D-Fuge devices presented here are capable of centrifugation of a wide variety of different solutions such as spinning down samples for biomarker applications and performing nucleotide extractions as part of a portable molecular lab setup. We introduce the design and proof-of-principle trials that demonstrate the utility of low-cost 3D-printed centrifuges for use in remote field biology and educational settings.

## Abstract 2

This Community Page article describes a low-cost 3D-printed centrifuge to enable sequencing in remote field conditions and lowering the barrier to synthetic biology research in high schools to broaden participation in hands-on STEM.

## Introduction

The centrifuge is an indispensable piece of equipment for laboratories, with general applications ranging from DNA isolation to clinical diagnostics. Yet, conventional centrifuges are often inaccessible outside of established lab settings (such as remote field sites), require a constant external power source, and can be prohibitively costly for STEM-focused programs. Progress has been made in the field of frugal science [1–6], a new approach towards making scientific tools more accessible and transportable, but there are many devices that remain to be developed or are currently in developmental stages. It is crucial to produce new low-cost devices to ensure increased access to scientific tools and expand scientific research without inhibition from costs and accessibility. Recently, 3D-printing technology has emerged as a convenient method for the rapid development and production of cost-effective scientific and diagnostic tools [7].

**Fig 1:**
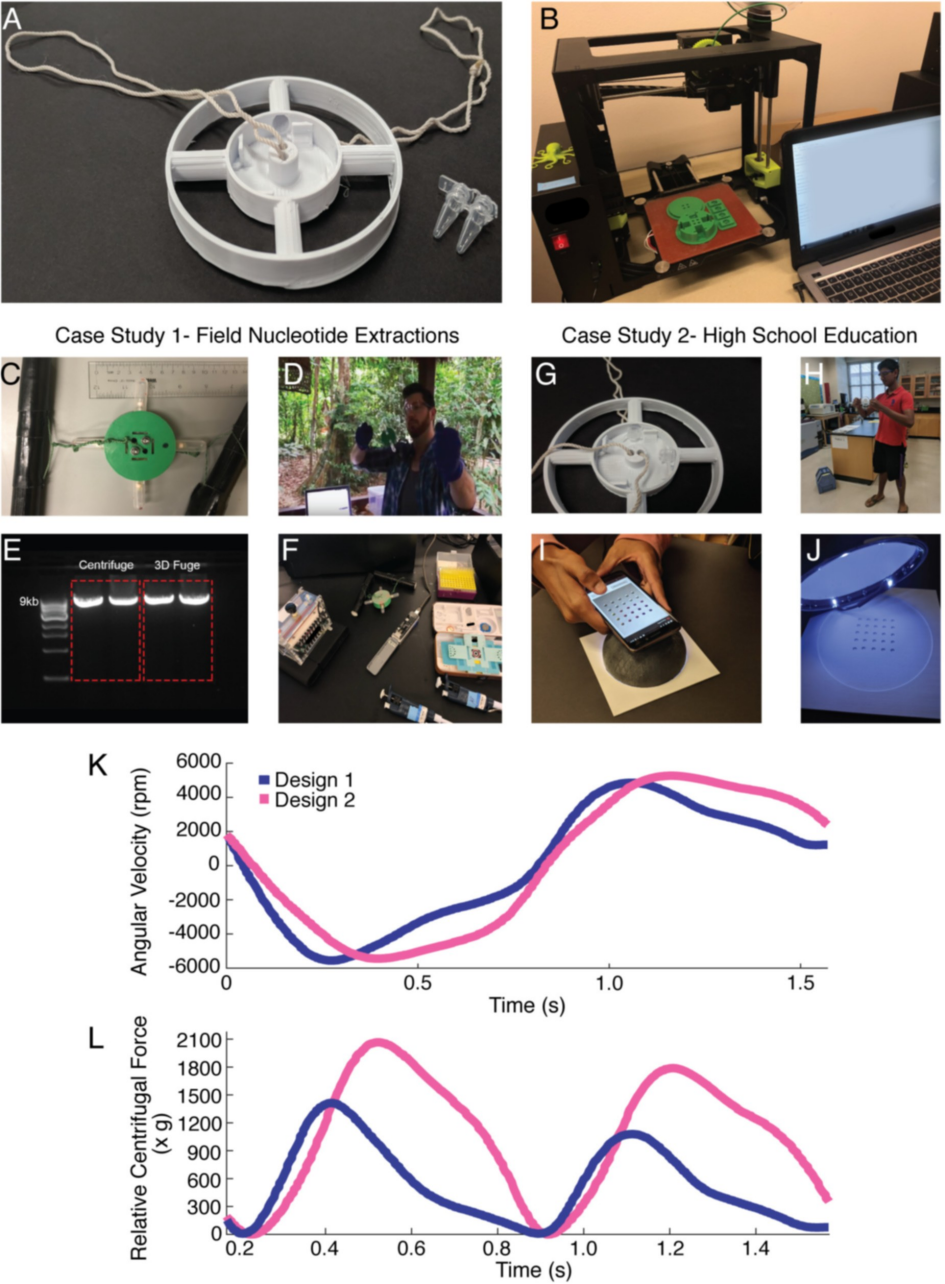
Overview of the 3D-Fuge. (A) Example of the 3D-Fuge utilized for Case Study 2, with 0.2 mL PCR tubes (VWR, USA) placed alongside for comparison. (B) Construction of the 3D-Fuge through 3D Printing. (C) 3D-Fuge utilized in Case Study 1 (rainforest in Peru), shown holding up to 4 spin-columns and flow-through tubes for nucleotide extractions. (D) Example usage of the 3D-Fuge in the field located in Southeastern Peru. (E) Comparison of long-range PCR results from human cheek swab nucleotide extractions using a standard benchtop centrifuge (left) and the 3D-Fuge (right). (F) Image of the portable lab setup, including the 3D-Fuge, the miniPCR (miniPCR) powered by an external battery pack, the MinION DNA sequencer (Oxford Nanopore Technologies, USA), and the Foldscope (Foldscope Instruments, USA). (G) Image of the 3D-Fuge utilized in Case Study 2 (high school in Atlanta, Georgia) shown holding PCR tubes (0.2 mL). (H) Usage of the 3D-Fuge in a high school. (I) Image of color output processing for data collection of chromoprotein expression. Phone captures the image of liquid culture pellets for RGB color analysis. (J) Sample illumination chamber utilized for standardized sample illumination. (K–L) Representative time evolution of the RPM and RCF of the 3D-Fuges over 2 cycles (counterclockwise and then clockwise inversion) utilized in each of the case studies. The peak RPM achieved is approximately 6,000 for both case studies. The above graphs illustrate the overall oscillatory motion of the 3D-Fuge, the change in angular velocity demonstrating the peak RPM over an interval, and the changing relative centrifugal force with a relative maximum of 2100*g* for Case 1, and 1,400*g* for Case 2, through the given average interval. Data for the graph can be found on GitHub (https://github.com/bhamla-lab/3D-fuge-PlOS-Biology-2019) in the file named Data File for 3D-Fuge Figures (2) under the sheet titled “Fig 1K–1L.” PCR, polymerase chain reaction; RCF, relative centrifugal force; RGB, Red-Green-Blue.

Here, we present the 3D-Fuge, a 3D-printed device based on the principles of the paperfuge [2] as a low-cost human-powered alternative to standard benchtop centrifuges. The paperfuge is a recently developed ultralow-cost (US$0.20), human-powered centrifuge that can be useful for applications including blood separation and disease diagnostics (such as anemia and malaria). Although the paperfuge is capable of centrifuging samples at speeds of up to 125,000 rpm and exerts centrifugal forces of 30,000*g*, it is limited by the sample volume it can hold (20 μL per capillary tube). The 3D-Fuge in this study addresses the paper-based centrifuge's limitation by expanding the liquid volume capacity (up to 2 mL) of samples, thereby enabling applications for workflows in molecular biology such as nucleotide extractions. It is capable of holding and spinning down 4 samples ranging from the size of capillary tubes and polymerase chain reaction (PCR) tubes to nucleotide extraction spin-column tubes, allowing for the centrifugation of a wide variety of different solutions. The production of this device is fairly inexpensive, is less than US$1.00 (Table 1), can be produced by anyone with access to a 3D printer, and can reach hand-powered centrifugation speeds up to 6,000 rpm or 2000*g* relative centrifugal force (RCF; Fig 1A, 1B, 1K and 1L; S1 Fig, S2 Fig, S1 Text). It is highly portable and requires no continuous access to electricity, making it easy to transport and utilize for various applications. Two different designs of the 3D-Fuge are presented: Design 1 is capable of holding spin-column tubes with a volume capacity of 2.0 mL (Fig 1A and 1C) to enable workflows for nucleotide extractions, and Design 2 is capable of holding PCR tubes with a volume capacity of 0.2 mL (Fig 1B and 1G) to enable workflows with bacterial cell pelleting.

**Table 1:** Summary of the 2 different 3D-Fuge designs. The total cost was calculated by combining the cost of the string used and 3D-printing material (see S1 Text for price details). The cost excludes the cost of the 3D printer and its associated parts. Cost is given in US Dollars. The files for the 3D-Fuge designs can be found on GitHub https://github.com/bhamla-lab/3D-fuge-PlOS-Biology-2019.

3D-Fuge Model	String Length (m)	Weight (grams)	Radius (mm)	Volume (mL)	RCF (× *g*)	Cost
Design 1	0.46–0.56	26.30	30	2.0	1,370	$0.72–$0.74
Design 2	1.04	13.50	45	0.2	2,070	$0.41

**Abbreviations:** RCF, relative centrifugal force.

To demonstrate the capability of this device to perform routine experiments without access to conventional laboratory equipment, we carried out nucleotide extractions under both lab and remote field conditions (Fig 1, Case 1), with yields comparable to conventional benchtop centrifuges. We then performed downstream experiments from the 3D-Fuge extractions, including long-range PCR amplification and real-time nanopore DNA sequencing. We also integrated this device with a novel chromoprotein analysis application by producing bacterial pellets for quantification as part of a high school STEM program experiment (Fig 1, Case 2). Through these studies, we demonstrate the usage of the 3D-Fuge in different parts of the world (from a rainforest in Peru to a public high-school in the United States) to validate its broad applicability. Due to its low cost and ease of use, the 3D-Fuge can be valuable for a range of areas, including field research, disease-screening in developing countries, and science education.

## Results

### Expanding field genomics through portable nucleotide extractions and nanopore sequencing

**Fig 2:**
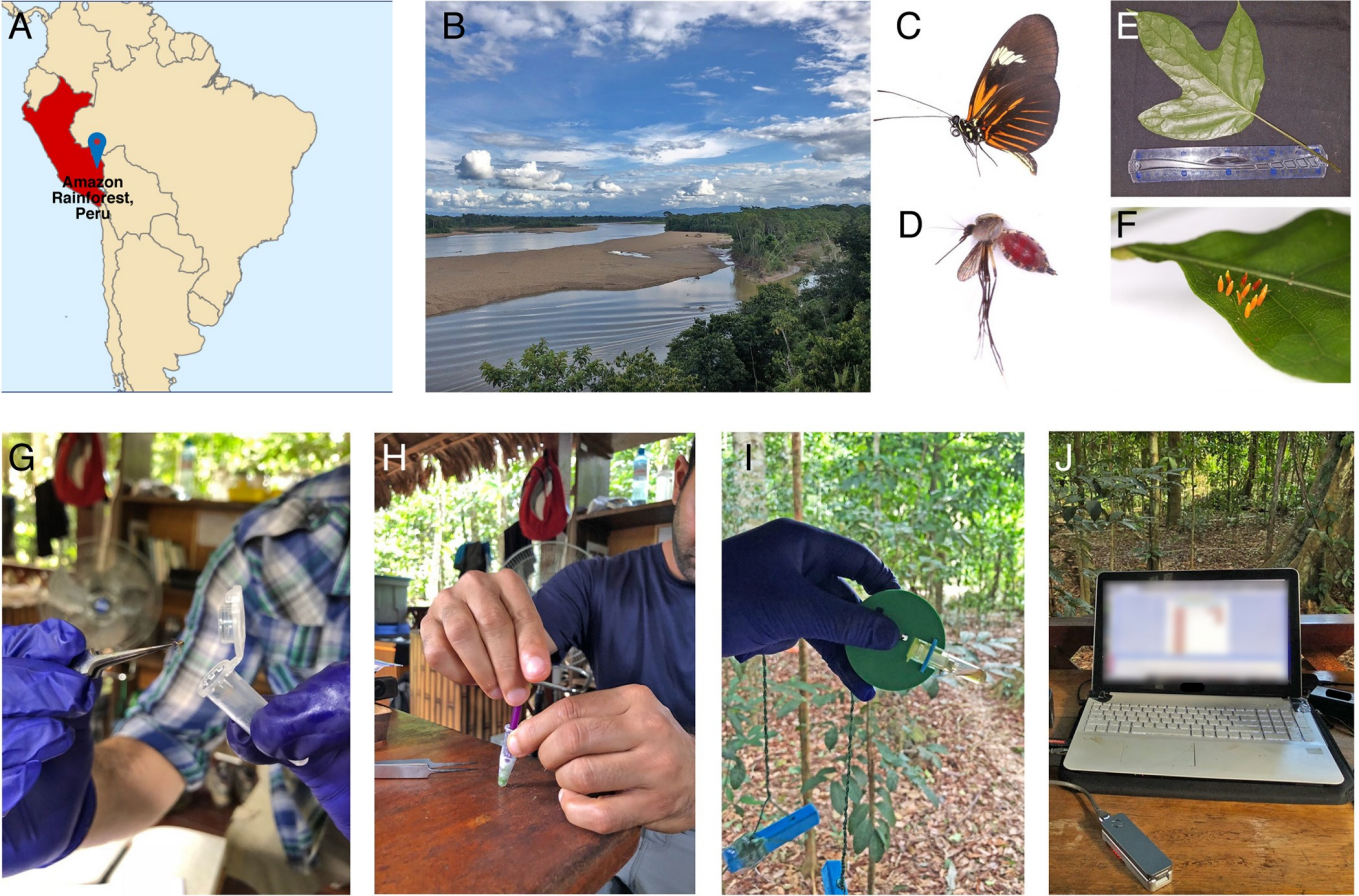
Overview of steps involved with sample collection, use of the 3D-Fuge for nucleotide extractions, and DNA sequencing in the Amazon rainforest. (A, B) Field site for the portable lab study located in Tamboapta, Peru. (C–F) Examples of specimens collected for *in situ* DNA sequencing, including (C) a butterfly, (D) a bloodfed mosquito, (E) a plant leaf, and (F) unknown insect eggs. (G, H) Tissue from specimens were homogenized and incubated in proteinase K and (I) the hand-powered 3D-Fuge was then used to perform DNA extraction and purification. (J) Purified DNA was subsequently used for downstream molecular steps including long-range PCR and real-time DNA sequencing on the MinION connected to a laptop. PCR, polymerase chain reaction.

Nucleotide extractions are a necessary first step for numerous molecular experiments, such as DNA sequencing projects, and often require centrifugation steps to separate and purify high-quality nucleic acids from the sample of interest. The ability to rapidly extract and purify nucleic acids with a low-cost hand-powered centrifuge can be useful for a wide range of molecular applications when one does not have access to conventional laboratory equipment, such as in the field or in resource-limited settings. Portable sequencing projects are already emerging in applied field settings, including real-time species or environmental sample identifications [8, 9], pathogen diagnostics [10, 11]), and metagenomics [12, 13], but most studies thus far have utilized benchtop centrifuges with external power sources to prepare samples. Therefore, to demonstrate the capability of performing nucleotide extractions in a remote environment outside of a conventional laboratory, the 3D-Fuge (Fig 2A–2C) was deployed during a biodiversity research expedition in Tambopata, Peru, at the Refugio Amazonas lodge (−12.865231, −69.409545; Fig 2A and 2B).

Before the expedition, we first compared DNA extractions in the lab using a standard benchtop centrifuge (Eppendorf, model 5415 D) and the hand-powered 3D-Fuge. A human cheek swab sample was collected and DNA extractions were carried out using the Quick-DNA Miniprep Plus Kit (Zymo Research, Irvine, CA) according to manufacturer's protocol. Eluted DNA yields were assessed using a Nanodrop, and the results between centrifuge strategies, such as nucleotide concentration and a 260/280 ratio, were comparable (S3 Fig). The quality of the DNA from both extractions was also sufficient to perform long-range PCR amplification of 9,000 bp fragments of the human mitochondrial genome (Fig 1E), indicating that the hand-powered 3D-Fuge was capable of performing nucleotide extractions without requiring lab infrastructure.

Next, while in the Peruvian Amazon, specimens such as whole insects and plant leaves were collected and preserved in 1.5 mL Eppendorf tubes containing DNA Shield (Zymo) for downstream processing (Fig 2C–2G). Molecular experiments in the field, including DNA extractions, long-range PCR, library preparation, and DNA sequencing, were performed using a highly miniaturized laboratory consisting of portable equipment [9]. The main components involved for this study include the 3D-Fuge, a small thermocylcer (miniPCR), and the MinION, a handheld nanopore-based sequencing device (Oxford Nanopore Technologies; Fig 1F).

As before, all DNA extractions in the field were carried out using the Quick-DNA Miniprep Plus Kit (Zymo). Specimens were homogenized using a pestle and incubated with proteinase K for 1 to 3 hours (Fig 2G and 2H). The 3D-Fuge for nucleotide extractions was designed specifically to fit and hold up to 4 standard 2 mL polypropylene spin-column tubes, which in this study were provided as part of the DNA isolation kit (S4A–S4C Fig). Samples were transferred to the spin-column tubes and placed into the 3D-Fuge, which was spun by hand at maximum speed for approximately 1 to 2 minutes for each centrifugation step, including sample extraction, purification, and elution (Fig 1D, Fig 2I). We were not able to quantify nucleotide extractions while in the field, so we chose to use 1 to 3 μL of eluted DNA from the 3D-Fuge for downstream long-range PCR reactions, which were performed using the Q5 Hot Start High-Fidelity 2X Master Mix (New England Biolabs, Ipswich, MA) and dual-indexed primers to amplify the ribosomal DNA (rDNA) cluster from plant and arthropod samples [14]. These PCR products were run on a gel to verify amplification after the expedition back at the lab, and the gel indicated that all 4 extractions from the 3D-Fuge were successful (S3A–S3D Fig). In the field, approximately 1 to 2 μL of PCR product for each of the samples were pooled, and the Oxford Nanopore Technologies SQK-LSK 108 library preparation was carried out according to manufacturer's protocol (ONT). The final library was run on the MinION, and rDNA amplicons were sequenced in real-time on a laptop in the field (Fig 2J). Raw sequence reads were generated on a laptop in the field, and a bioinformatics pipeline was run to demultiplex samples and create a consensus sequence for each sample [14].

For the butterfly (Fig 2C), an rDNA consensus sequence of 4,658 bp in length was generated. The closest BLAST hit in the LepBase.org database was to *Heliconius*, a likely match based on morphology and genetic data. The bloodfed mosquito (Fig 2D) extraction yielded a consensus sequence 3,931 bp in length. BLAST and distance of tree results in the NCBI database yielded the closest match to a species in the genus *Psorophora* (S3A–S3D Fig). The plant specimen (Fig 2E) yielded a final consensus sequence 3,263 bp, and the BLAST result was the closest match to species in the nightshade family Solanaceae, which was expected based on morphological identification by a botanist (Varun Swamy, personal communication). Finally, the eggs (Fig 2F) belonged to an unknown species of insect. The consensus sequence generated was 4,028 bp, and BLAST results yielded the closest match to a butterfly species in the family Pieridae. The host plant was not detected in the sequence data with the insect eggs, but we did pick up a fungal sequence as well with a BLAST hit to the genus *Zymoseptoria*, which may have been an environmental sample on the leaf. Overall, the portable lab equipment enabled detection of specimens through rapid *in situ* DNA barcoding, and use of the hand-powered 3D-Fuge demonstrated the feasibility of extracting high-quality nucleic acids from samples in a remote tropical environment such as the Amazon rainforest.

### Increasing accessibility to synthetic biology research in high school settings

**Fig 3:**
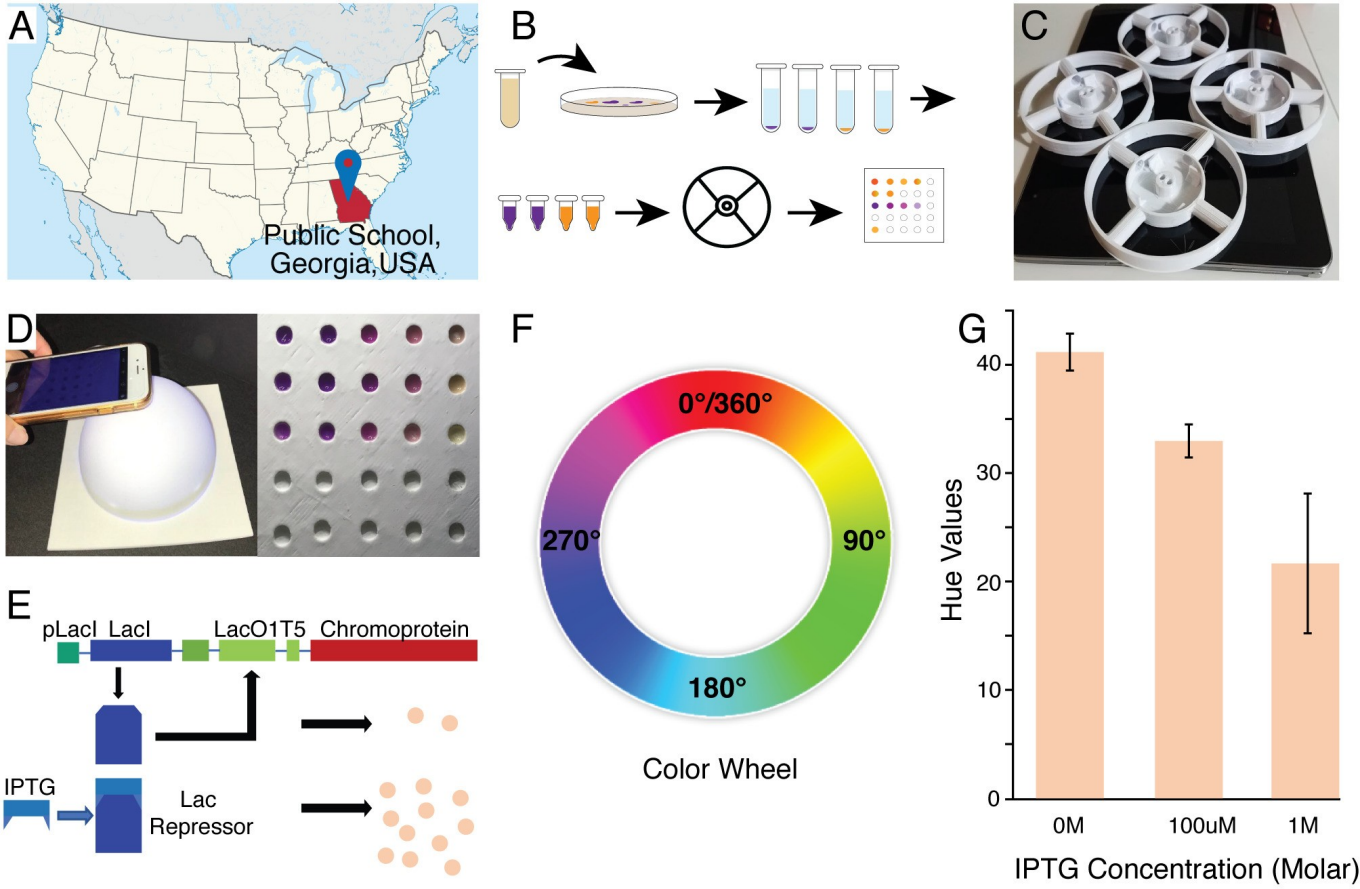
Analysis of chromoprotein expression in high-school. (A) Location of high school on map of the United States. (B) Workflow schematic for color analysis, beginning with transformation of the plasmid and plating, growth of liquid cultures, isolation of small portion of culture, centrifugation with 3D-Fuge, and sample illumination chamber and color picker for measurements. (C) Images of 3D-Fuges utilized in experiments. (D) Image of sample illumination chamber and phone used for capturing the image (Left). Image of view of chromoprotein-expressing bacterial pellets in sample illumination chamber (Right). This image does not represent the pellets utilized for measurements indicated in Fig 3G. (E) Diagram for regulation of chromoprotein expression in plasmid construct. (F) Color wheel utilized for hue value measurements. As darker shades occur counterclockwise around the color wheel, darker colors correlate with a smaller hue value. (G) Hue value measurements for Scrooge Orange chromoprotein at varying IPTG concentrations. As expected, increasing IPTG concentrations results in smaller hue values, indicating successful analysis of color expression using the 3D-Fuge and sample illumination chamber. SD, *N* = 3 technical replicates. Data for the graph can be found on GitHub (https://github.com/bhamla-lab/3D-fuge-PlOS-Biology-2019) in the file named Data File for 3D-Fuge Figures (2) under the sheet titled “Fig 3G.” IPTG, Isopropyl β-D-1-thiogalactopyranoside.

Reporter proteins are a quintessential part of synthetic biology [15], from identifying successful expression of genetic constructs to acting as biomarkers for diagnostic applications[16, 17]. Chromogenic proteins (or chromoproteins) are examples of such reporters capable of producing a color visible to the naked eye, unlike fluorescent proteins, which are more commonly utilized [18, 19]. Although fluorescent proteins are often quantified using plate readers, chromoproteins rely on the Red-Green-Blue (RGB) and Hue-Saturation-Luminance (HSL) color spaces for measurements [18], specifically with a focus on the corresponding hue value. In order to successfully obtain the hue values for bacterial samples expressing the reporter proteins, they must be concentrated into pellets to standardize the color values. Here, we report the usage of the 3D-Fuge for centrifugation of bacterial pellets for corresponding measurements using a sample illumination chamber and RGB color analysis in a public high school environment in Georgia (Fig 3A and 3B, Fig 1G–1J). This iteration (Fig 3C, S5A–S5C Fig) is capable of centrifuging four 0.2 mL PCR tubes at speeds of up to 6,000 rpm, and to demonstrate its functionality, we centrifuge chromoprotein-expressing bacteria for color measurements.

The Isopropyl β-D-1-thiogalactopyranoside (IPTG)-inducible chromoprotein plasmid constructs (obtained from ATUM Bio, 2018; Fig 3E) were transformed into DH5a *Escherichia coli* and subsequently inoculated into liquid cultures with varying concentrations of IPTG. Following the growth phase, liquid cultures were allowed to settle, and a portion of the settled particulate was centrifuged using the 3D-Fuge for 5 minutes. The supernatants were discarded, and the obtained cellular pellets were placed into the sample illumination chamber to capture an image with a phone (Fig 3D). The image was then processed to extract the RGB and/or HSL values to determine the hue of the bacterial pellet.

The chamber was utilized to ensure standardized white illumination of the sample and facilitate the capturing of an image through the opening at the top (Fig 3D). The corresponding images of samples were then processed through an RGB color-analysis tool to obtain the hue values, signifying the level of chromoprotein expression present. Using this measurement scale, increased chromoprotein expression would be represented by a lower value whereas decreased chromoprotein expression would be represented by a higher value. This is due to the orientation of the RGB color space in which specific colors increase in darkness counterclockwise (or in order of decreasing values; Fig 3F). From the trials conducted, the expected decrease in hue values can be seen with increase in IPTG concentration (Fig 3G), indicating that the 3D-Fuge was able to successfully centrifuge the liquid culture samples and obtain bacterial pellets reflecting the levels of chromoprotein expression. We thereby demonstrate the applications of the 3D-Fuge in enabling chromoprotein analysis for synthetic biology education in high schools, opening up new possibilities for low-cost biomarker analyses that do not require expensive plate readers for quantification.

## Discussion

Developments in portable nucleotide sequencing hold great promise for fields such as human health applications, metagenomics, agriculture, and molecular taxonomy [20]. Third-generation portable sequencing instruments, such as the MinION (ONT), require high-quality input DNA or RNA [21]. We therefore set out to obtain purified DNA extractions without access to conventional laboratory equipment while in a remote tropical rainforest for a real-time DNA barcoding study, which can allow for the identification of specimens via DNA amplification and sequencing [22]. For the purpose of sequencing, the 3D-Fuge was designed to hold DNA spin-column tubes (2 mL). Centrifuge-free methods for sequencing that use cellulose-based paper have been demonstrated in the past [23]. However, for samples that are difficult to isolate nucleotides from and perform certain experiments such as long-range PCR or nanopore sequencing, centrifugation is useful for producing high-quality nucleic acid extracts through repeated washing steps and elutions. We found that DNA extractions using the 3D-Fuge were of sufficient quality compared with a standard benchtop centrifuge, with extracts used for downstream long-range PCR amplification of products from around 3,500 bp (spanning the ribosomal cluster) to around 9,000 bp (spanning about half of the human mitochondrial genome) in length (Fig 1, Fig 2).

One limitation to the current 3D-Fuge design is that it can hold up to 4 spin-column tubes at once, which means increasing radius and weight to accommodate additional tubes may reduce the maximum speed and increase muscle fatigue by the user [2]. This restricts the number of samples that can be processed simultaneously, but due to the low-cost nature of the 3D-printed device, several units can be produced and additional users could perform hand-powered centrifugation steps in parallel. Another limitation to the 3D-Fuge is the rotational speed it can achieve, which is attributed to its increased weight as well as use of a more robust polymeric material (PLA) for construction. For example, the paperfuge weighs 2 g, whereas the 3D-Fuge weighs 20 g (exact weights for each design can be found in Table 1), almost 10 times more. Thus, as expected, there is a trade-off in the rotation speed 20,000 RPM (paperfuge) versus 6,000 RPM (3D-Fuge), which is necessary to achieve the larger volume capacity needed for the molecular biology applications demonstrated in this work.

## Conclusions

The field of frugal science is helping to develop new low-cost portable tools and applications, such as the ability to perform real-time diagnostics in remote environments, and enabling greater access to those with an interest in scientific devices, such as high school students. Here we introduce the 3D-Fuge, a 3D-printed device capable of centrifugation of a wide variety and volumes of solutions, such as spinning down samples for biomarker applications and performing nucleotide extractions as part of a portable molecular lab setup. Overall, we hope that the design and proof-of-principle trials presented here will stimulate others to continue research into the development of low-cost scientific devices and that the 3D-Fuge will be valuable to a range of users including students, labs in resource limited settings, and field researchers.

## Supporting information

S1 Fig RPM values of 3D-Fuges. Shows the RPM of both designs over a cycle of 5 runs. Both designs have similar peak RPM at around 6,000; however, they have slightly different periods of revolution. The data shows reproducible rpm cycles with time. Data for the graph can be found on GitHub (https://github.com/bhamla-lab/3D-fuge-PlOS-Biology-2019) in the file named Data File for 3D-Fuge Figures (2) under the sheet titled “S4 and S5 Figs.”
(TIF)

S2 Fig RCF values of 3D-Fuges. Shows the RCF values of both designs over a cycle of 5 runs. Although both the designs have the same rpm values, Design 1 has a smaller g-force due to its smaller radius (see Table 1). Data for the graph can be found on GitHub (https://github.com/bhamla-lab/3D-fuge-PlOS-Biology-2019) in the file named Data File for 3D-Fuge Figures (2) under the sheet titled “S4 and S5 Figs.” RCF, relative centrifugal force.
(TIF)

S3 Fig Nucleotide extractions with 3D-Fuge. (A) Components and 3D-printed parts of the 3D-Fuge. (B) Comparison of human cheek swab DNA extractions using a conventional laboratory bench top centrifuge (left) and the 3D-Fuge (right) with their respective Nanodrop DNA quantifications. Long-range mitochondrial PCR products using these extracts can be found in Fig 1E. (C) Gel electrophoresis of samples that were extracted in the field using the 3D-Fuge and subsequently PCR amplified with ribosomal DNA primers (left to right: Solanaceae, *Heliconius* butterfly, mosquito, and butterfly eggs). (D) NCBI distance of tree results from a consensus sequence generated in the field from the bloodfed mosquito sample. NCBI, National Center for Biotechnology Information; PCR, Polymerase chain reaction.
(TIF)

S4 Fig 3D CAD model for the 1.5 mL 3D-Fuge. (A) Bird's eye view of the main piece for the 3D-Fuge including its dimensions. (B) Bottom-up view of the 3D-Fuge as well as its dimensions. (C) Connector piece(s) dimensions. CAD, Computer-aided design.
(TIF)

S5 Fig 3D CAD model for the 0.2 mL 3D-Fuge. (A) Top-down view of the 3D-Fuge and its dimensions. (B) Bottom-up view of the 3D-Fuge and its dimensions. (C) Bird's eye view of the 3D-Fuge including its dimensions. CAD, Computer-aided design.
(TIF)

S1 Text Methods and materials. Detailed protocols for chromoproteins transformation, 3D-Fuge design and materials, nucleotide extractions, and high-speed video analysis to estimate spinning speed of 3D-Fuges.
(PDF)

## Abbreviations

BLAST: Basic Local Alignment Search Tool
HSL: Hue-Saturation-Luminance
NCBI: National Center for Biotechnology Information
PCR: Polymerase chain reaction
PLA: polymeric material
RCF: relative centrifugal force
RPM: revolutions per minute
rDNA: ribosomal DNA
RGB: Red-Green-Blue
STEM: Science Technology Engineering and Math
