# Supplementary figures and images for: A 3D-printed hand-powered centrifuge for molecular biology
Source: PLoS Biol. 2019 May 21;17(5):e3000251. doi: 10.1371/journal.pbio.3000251 (PMC6528969; doi:10.1371/journal.pbio.3000251)

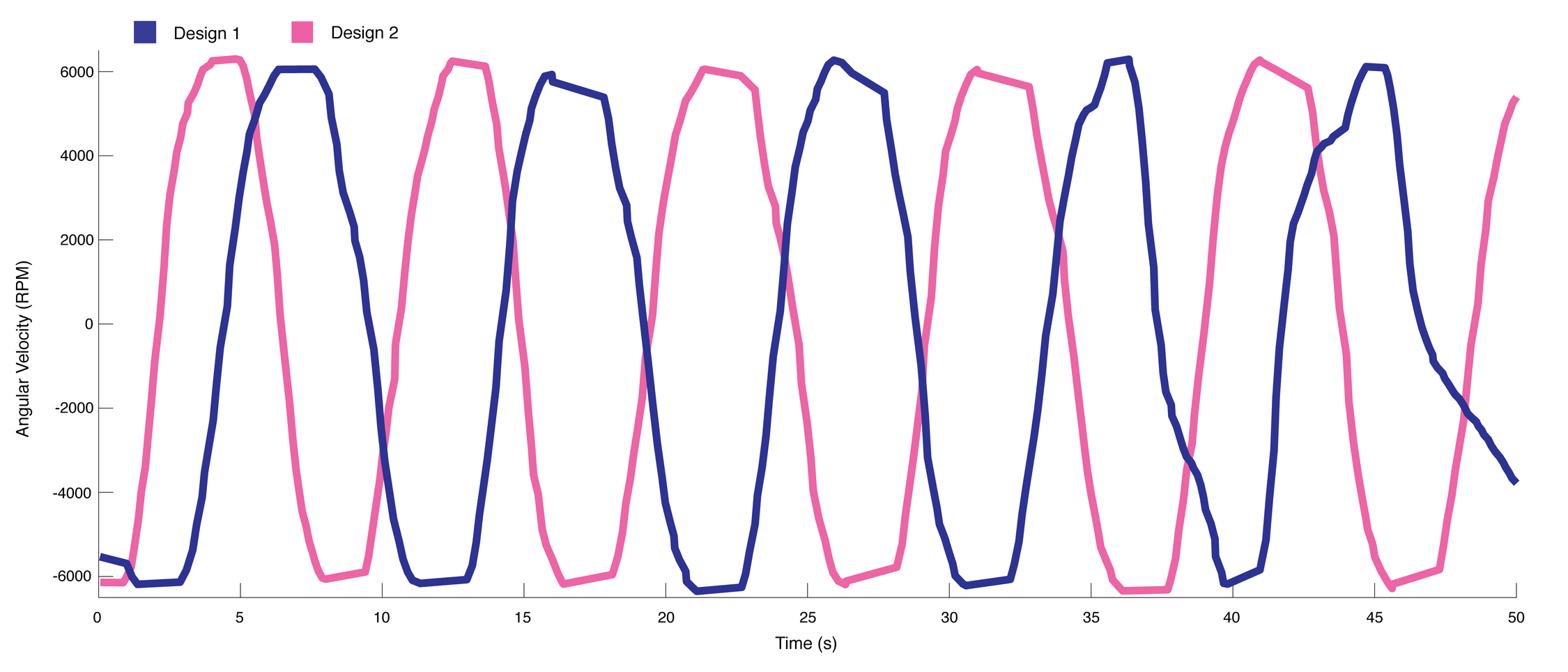

Supplement: S1 Fig — Shows the RPM of both designs over a cycle of 5 runs. Both designs have similar peak RPM at around 6,000; however, they have slightly different periods of revolution. The data shows reproducible rpm cycles with time. Data for the graph can be found on GitHub (https://github.com/bhamla-lab/3D-fuge-PlOS-Biology-2019) in the file named Data File for 3D-Fuge Figures (2) under the sheet titled “S4 and S5 Figs.” (TIF) [file pbio.3000251.s001.tif]

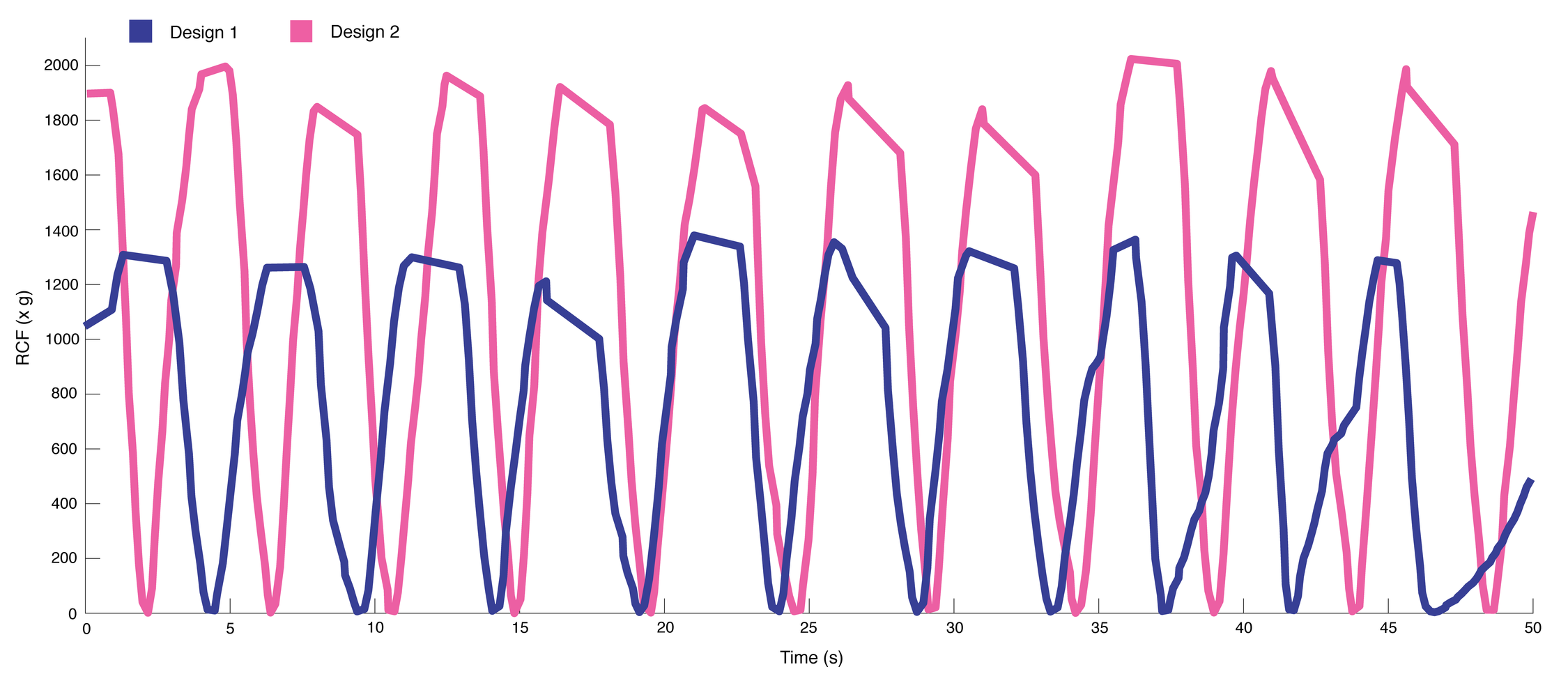

Supplement: S2 Fig — Shows the RCF values of both designs over a cycle of 5 runs. Although both the designs have the same rpm values, Design 1 has a smaller g-force due to its smaller radius (see Table 1). Data for the graph can be found on GitHub (https://github.com/bhamla-lab/3D-fuge-PlOS-Biology-2019) in the file named Data File for 3D-Fuge Figures (2) under the sheet titled “S4 and S5 Figs.” RCF, relative centrifugal force. (TIF) [file pbio.3000251.s002.tif]

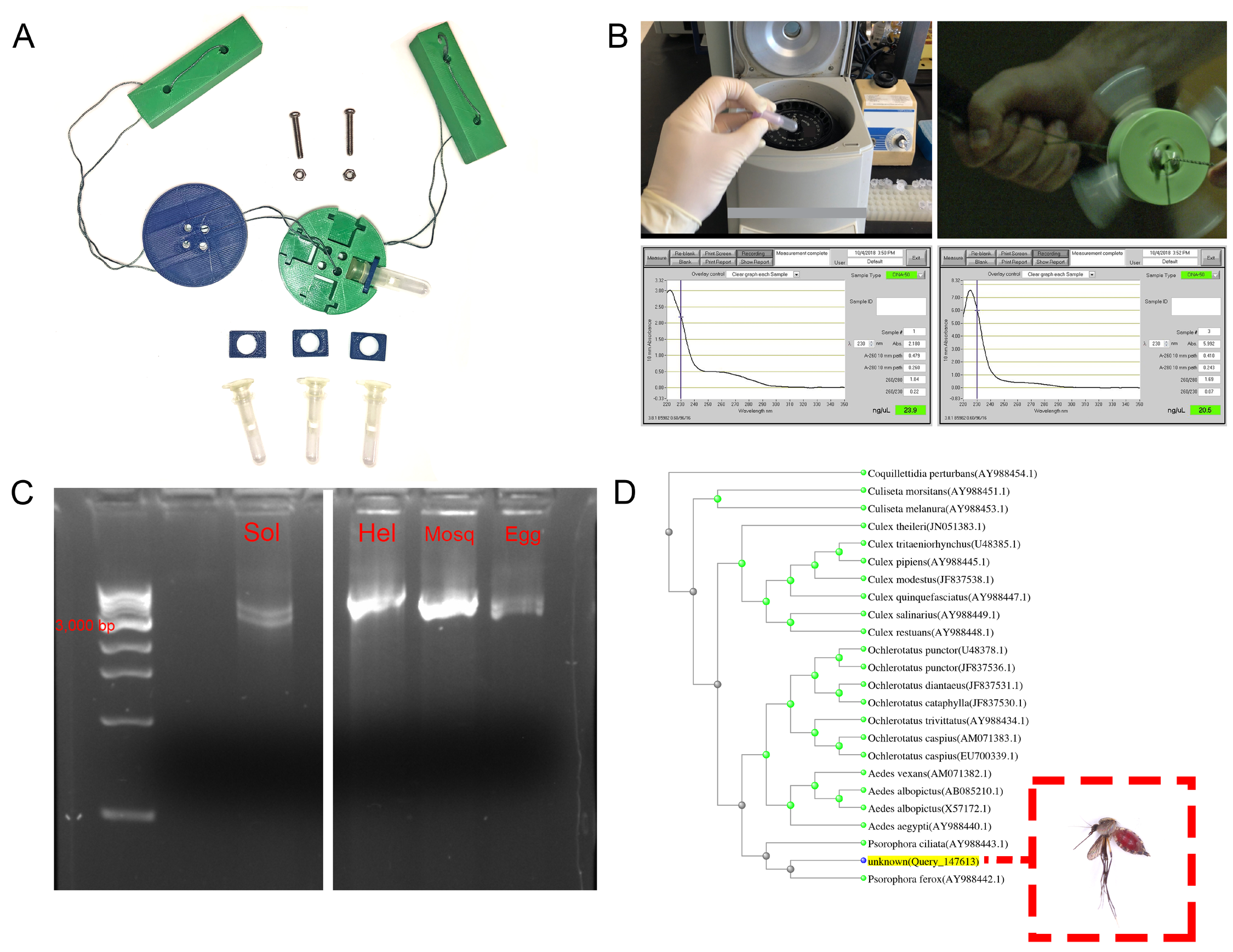

Supplement: S3 Fig — (A) Components and 3D-printed parts of the 3D-Fuge. (B) Comparison of human cheek swab DNA extractions using a conventional laboratory bench top centrifuge (left) and the 3D-Fuge (right) with their respective Nanodrop DNA quantifications. Long-range mitochondrial PCR products using these extracts can be found in Fig 1E. (C) Gel electrophoresis of samples that were extracted in the field using the 3D-Fuge and subsequently PCR amplified with ribosomal DNA primers (left to right: Solanaceae, Heliconius butterfly, mosquito, and butterfly eggs). (D) NCBI distance of tree results from a consensus sequence generated in the field from the bloodfed mosquito sample. NCBI, National Center for Biotechnology Information; PCR, Polymerase chain reaction. (TIF) [file pbio.3000251.s003.tif]

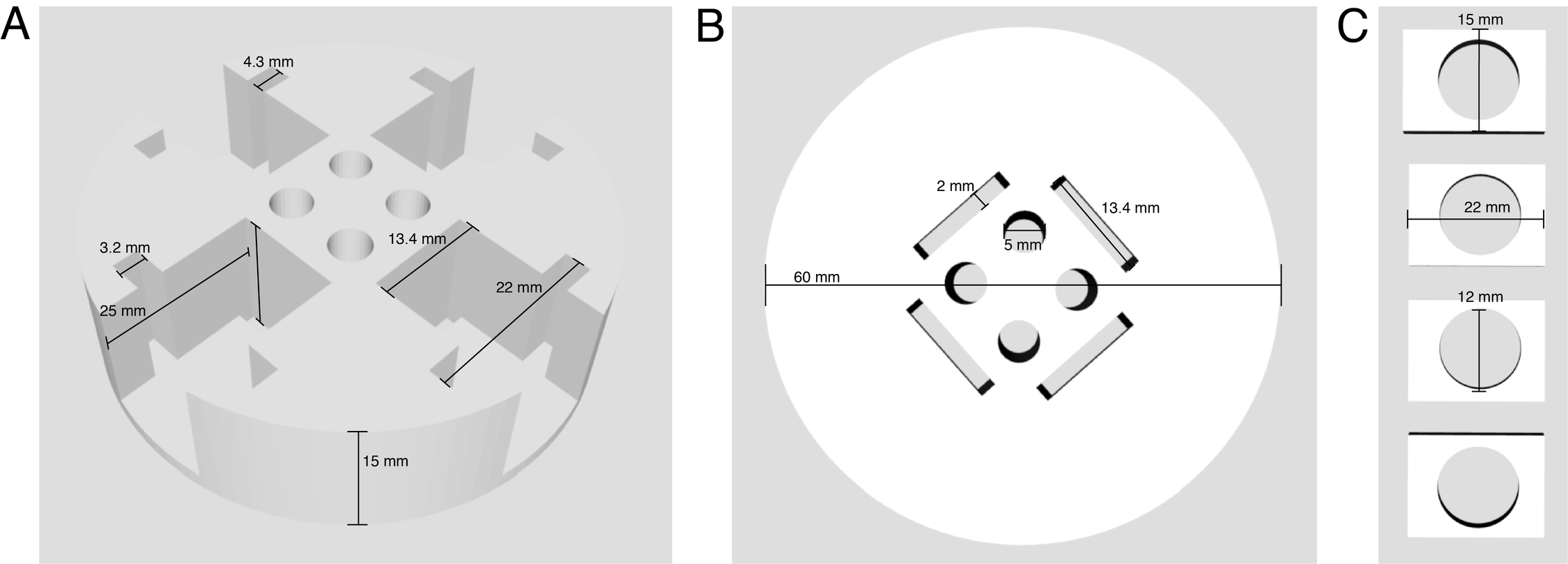

Supplement: S4 Fig — (A) Bird's eye view of the main piece for the 3D-Fuge including its dimensions. (B) Bottom-up view of the 3D-Fuge as well as its dimensions. (C) Connector piece(s) dimensions. CAD, Computer-aided design. (TIF) [file pbio.3000251.s004.tif]

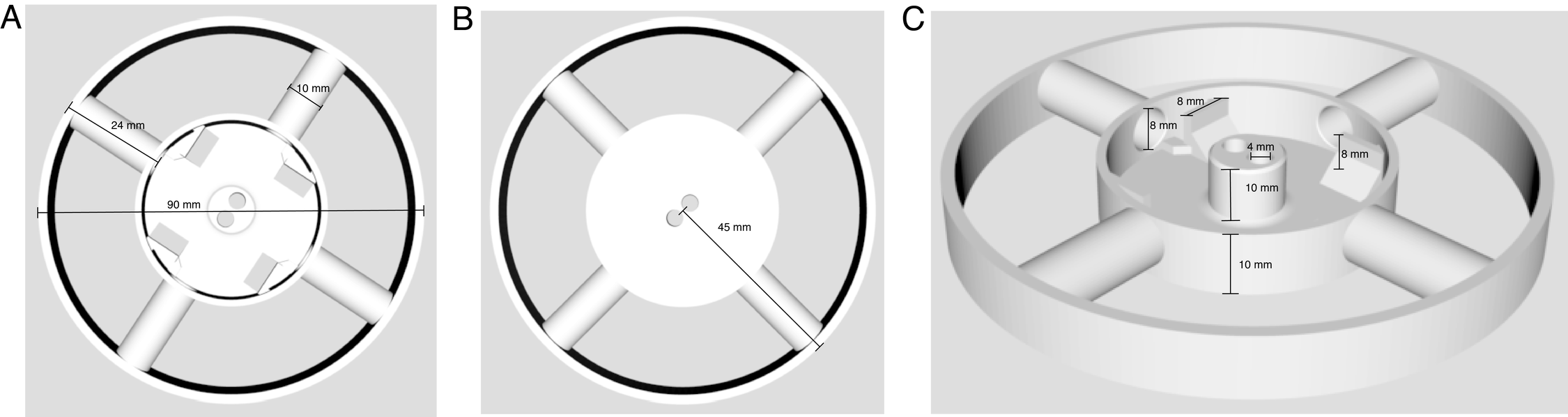

Supplement: S5 Fig — (A) Top-down view of the 3D-Fuge and its dimensions. (B) Bottom-up view of the 3D-Fuge and its dimensions. (C) Bird's eye view of the 3D-Fuge including its dimensions. CAD, Computer-aided design. (TIF) [file pbio.3000251.s005.tif]
