# Supplementary material for: A 3D-printed hand-powered centrifuge for molecular biology
Source: PLoS Biol. 2019 May 21;17(5):e3000251. doi: 10.1371/journal.pbio.3000251 (PMC6528969; doi:10.1371/journal.pbio.3000251)
Supplement: S1 Text — Detailed protocols for chromoproteins transformation, 3D-Fuge design and materials, nucleotide extractions, and high-speed video analysis to estimate spinning speed of 3D-Fuges. (PDF) [file pbio.3000251.s006.pdf]

# Supplementary Information - A 3D-printed hand-powered centrifuge for molecular biology

Gaurav Byagathvalli,[1, *] Aaron F. Pomerantz,[2, *] Soham Sinha,[3] Janet Standeven,[1] and M. Saad Bhamla[3, †]

[1] *Lambert High School, 805 Nichols Rd, Suwanee, GA, 30024, USA*
[2] *Department of Integrative Biology, University of California, Berkeley, CA, USA*
[3] *School of Chemical & Biomolecular Engineering, Georgia Institute of Technology, 311 Ferst Drive NW, Atlanta, GA 30332, USA*

## CONTENTS

I. Supplementary Movies 1
A. S1: 3D-Fuge: a 3D-Printed Hand-Powered Centrifuge 1

II. Supplementary Materials and Methods 1
A. Chromoprotein Transformation and Analysis 1
B. 3D-Fuge Design and Materials 1
C. Nucleotide Extractions 2
D. High-Speed Video Analysis 2

III. References 2

References 2

IV. Supplementary Figures 2

## I. SUPPLEMENTARY MOVIES

### A. S1: 3D-Fuge: a 3D-Printed Hand-Powered Centrifuge

Short video demonstrating the usage and applications of the 3D-Fuge both in field conditions and within high schools. The video can be viewed on YouTube at: https://www.youtube.com/watch?v=1yhtDpJqz8Q&feature=youtu.be.

## II. SUPPLEMENTARY MATERIALS AND METHODS

### A. Chromoprotein Transformation and Analysis

The chromoprotein plasmid construct (Scrooge Orange) was purchased from the corporation ATUM Bio (ATUM, 2018) to test in the competent *E. coli* cells. The samples obtained from ATUM Bio were hydrated in 10 $\mu$L of ddH20, and transformed into New England Biolabs DH5a *E. coli* using the NEB High Efficiency Transformation Protocol. The resulting transformation solutions were plated onto LB agar containing the antibiotic carbenicillin. Single colonies from the sample plate were diluted into 40 $\mu$L of water, and 1 $\mu$L of the colony dilution was inoculated into three different 5 mL LB broth liquid cultures with 5 $\mu$L of carbenicillin (100 $\mu$g/ml). These were induced with IPTG at concentrations of 0 M, 100 $\mu$M and 1 mM respectively. Liquid cultures grown for 24 hours in an 37° Celsius incubator set to shake at 170 rpm, and then removed from the incubator and allowed to sit undisturbed for 1 hour. 75 $\mu$L of the settled particulate was transferred into a 0.2 mL PCR tube and centrifuged for 5 minutes using the 3D-Fuge. The supernatant was discarded and the bacterial pellets were placed into the sample illumination chamber for measurements. An image captured with a phone and an RGB color-analysis tool (https://imagecolorpicker.com) were utilized to determine the hue values of each bacterial pellet [1]. These were then compared with the concentration of IPTG induction for analysis. As the color analysis tool uses a single pixel to determine the hue value, the pixel selected was representative of chromoprotein expression wherein the region within the bacterial pellet containing visible color expression (in the case of induced samples) contained the selected pixel.

### B. 3D-Fuge Design and Materials

In Case Study 1, one male individual (age 29) utilized a 3D-Fuge capable of holding microcentrifuge tubes as well as a combination of spin columns and flow-through tubes. In Case Study 2, four identical 3D-Fuges were utilized for the centrifugation of the chromoprotein samples. All devices were printed using a custom-built 3D Printer and CAD files modified for the inclusion of tube holders to ensure PCR tubes are securely placed in the device.

The 3D-Fuge design files can be found on Github https://github.com/bhamla-lab/3D-fuge-PlOS-Biology-2019 and example models with dimensions can be found in SI Fig 2A-C and SI Fig 3A-C. The design for nucleotide spin column extractions consists of a base that is 60 mm in diameter, a top that can fit over the base with two screws, and four rectangular holders that slide into grooves in the base that each hold one spin column tube in place (SI Fig1A). Design 1 3D-Fuges were printed using either

---

* authors contributed equally
† Please address correspondence to M.S.B: (saadb@chbe.gatech.edu)

a Lulzbot Mini or a Lulzbot Taz with Polylite (PLA) 2.85 mm filament. This PLA filament was purchased for approximately $25 as of March 2018. Design 2 3D-Fuges were printed using Zyltech 3D Printing Filament PLA 1.75 mm 1 kg/2.2 lbs. This PLA filament was purchased for approximately $20.

The string used in this study was the Dorisea Extreme Braid 500 lb 2.0 mm Fishing Line, purchasing 300m at a cost of approximately $40 as of March 2018. String lengths were maintained at a standard 104 cm, and 3D-Fuges were coiled 50 times prior to centrifugation. We also note that fishing-line string breaks can occur during centrifugation and insist the use of safety goggles when using the 3D-Fuge, as the device and components can reach high speeds. For Design 2, tape can additionally be used to secure the PCR tubes in place along with the existing latch for added protection.

### C. Nucleotide Extractions

The 3-D centrifuge was used to perform DNA extractions using the Quick-DNA Miniprep Plus kit (Zymo Research) according to the protocol. For DNA extraction comparisons in the lab, a benchtop centrifuge (Eppendorf model 5414 D) was used and each centrifugation step was performed at 13,000 rpm for 1 minute. Solid tissues collected in the field were lysed and added to a microcentrifuge tube with 95 $\mu$L of water, 95 $\mu$L of solid tissue buffer, and 10 $\mu$L of proteinase K for 1-3 hours. 400 $\mu$L of Genomic Binding Buffer was added to the supernatant and transferred to a Zymo-spin Column in a collection tube. The tubes were placed into the 3D-Fuge and spun by hand for approximately one to two minutes. The flow through was discarded, then 400 $\mu$L of DNA pre-wash buffer was added and centrifuged using the 3D-Fuge. Two rounds of washing steps were performed using gDNA Wash Buffer with 3D-Fuge centrifugation steps for one minute each followed by discarding of the flow through. 30 $\mu$L of DNA elution buffer was added and a final centrifugation step was carried out in a clean 1.5 $\mu$L tube. The final purified DNA was used for downstream molecular experiments, including polymerase chain reaction. Primers for long-range PCR were designed to amplify mitochondrial DNA (Fig 1F)[2] or ribosomal DNA [3]. Amplicons were pooled and sequenced on the MinION platform (Oxford Nanopore Technologies)[3]. The sequencing data can be found on GitHub.

### D. High-Speed Video Analysis

The angular velocity of the 3D-Fuge was determined through high-speed video tracking. A marker was placed on the centrifuge body, and was spun at maximum force by user which was captured using high-speed video at 1,057 frames per second (fps). Tracker (software) was used to track the marker through the high speed video frame by frame [4]. The angular velocity of 5 full cycles were tracked for both designs. The raw angular velocity data was smoothed using a LOESS function with a factor 0.02, and RPM and RCF were plotted in Figures S4 and S5. This is a second trial of the data shown in the main manuscript in Figure 1K, which is a representative of this data from a different trial. Average RPM and RCF is given as the square root mean of the peak rpm. Code for high-speed analysis can be found on `https://github.com/bhamla-lab/3D-fuge-PlOS-Biology-2019`.

## III. REFERENCES


[1] "Image Color Picker," .

[2] A. C. Clarke, BMC Genomics (2014), 10.1186/1471-2164-15-68.

[3] H. Krehenwinkel, A. Pomerantz, J. B. Henderson, S. R. Kennedy, J. Y. Lim, V. Swamy, J. D. Shoobridge, N. H. Patel, R. G. Gillespie, and S. Prost, bioRxiv (2018), 10.1101/358572.

[4] D. Brown, "Tracker," (2019).


## IV. SUPPLEMENTARY FIGURES

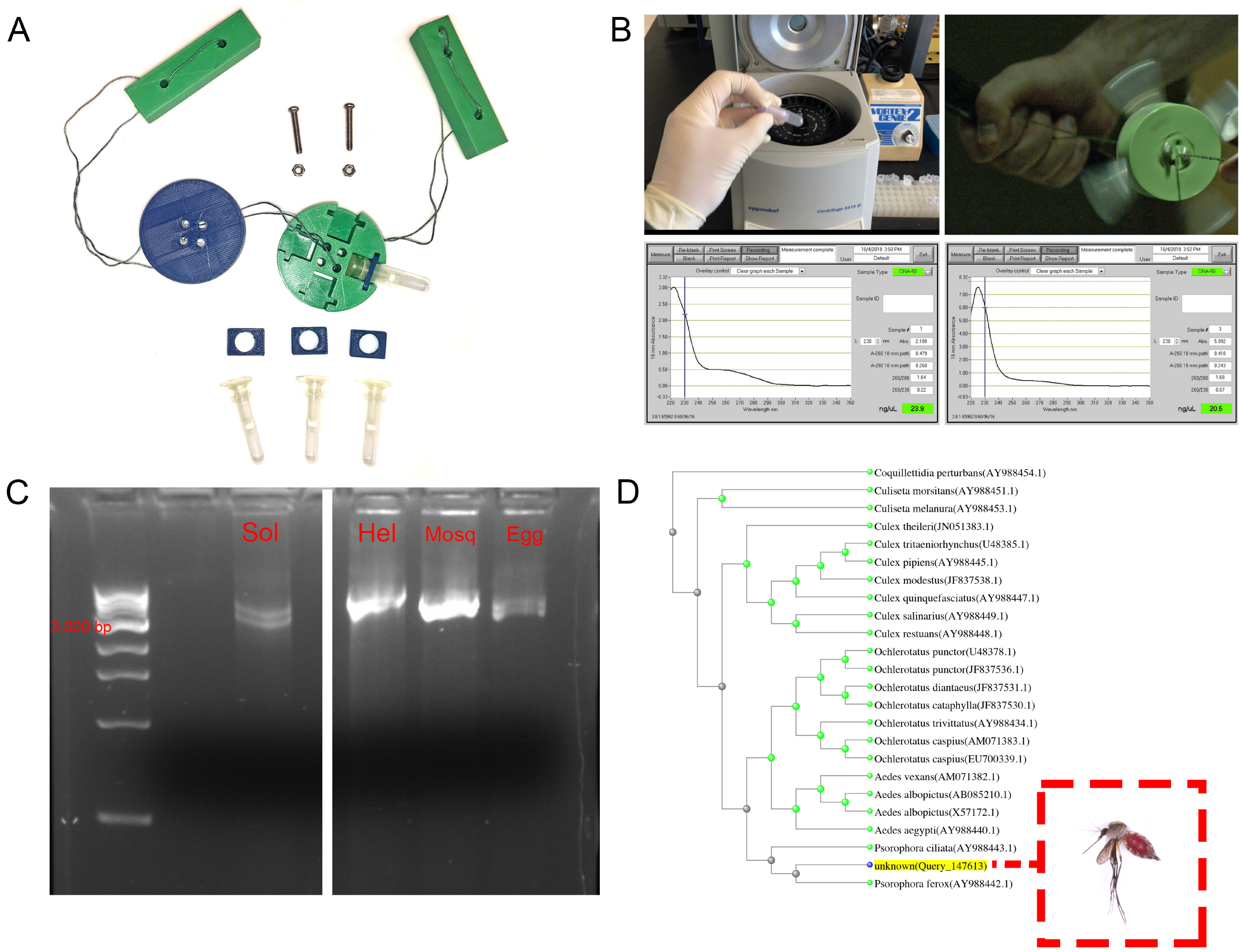


FIG. S1. **Nucleotide Extractions with 3D-Fuge.**(A) Components and 3D printed parts of the 3D-Fuge. (B) Comparison of human cheek swab DNA extractions using a conventional laboratory bench top centrifuge (left) and the 3D-Fuge (right) with their respective Nanodrop DNA quantifications. Long-range mitochondrial PCR products using these extracts can be found in Figure 1E. (C) Gel electrophoresis of samples that were extracted in the field using the 3D-Fuge and subsequently PCR amplified with ribosomal DNA primers (left to right: Solanaceae, Heliconius butterfly, Mosquito, and Butterfly eggs). (D) NCBI distance of tree results from a consensus sequence generated in the field from the bloodfed mosquito sample.

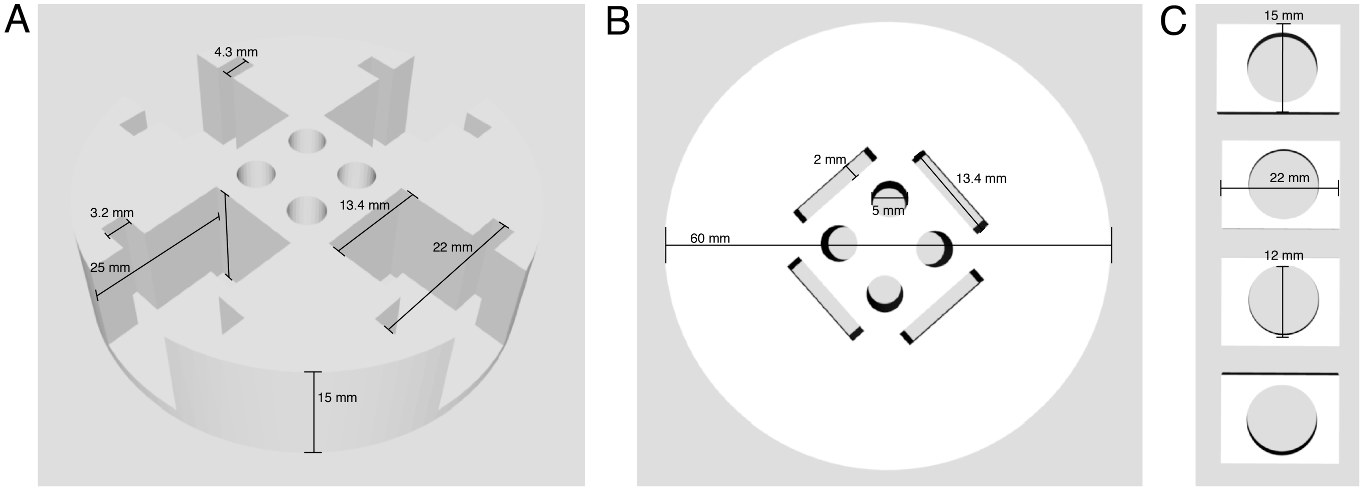


FIG. S2. **3D-Model for the 1.5 mL 3D-Fuge.**(A) Bird's eye view of the main piece for the 3D-Fuge including its dimensions. (B) Bottom-up view of the 3D-Fuge as well as its dimensions. (C) Connector piece(s) dimensions.

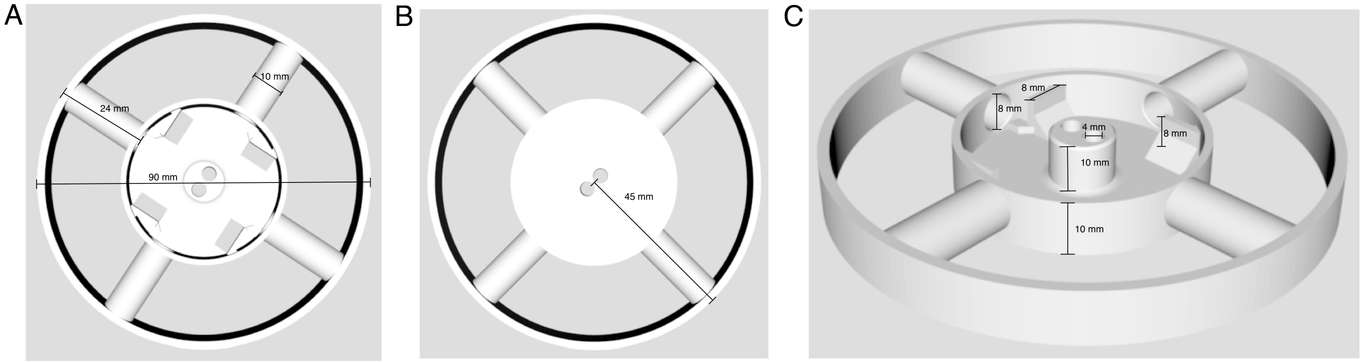


FIG. S3. **3D-Model for the 0.2 mL 3D-Fuge.**(A) Top-down view of the 3D-Fuge and its dimensions. (B) Bottom-up view of the 3D-Fuge and its dimensions. (C) Bird's eye view of the 3D-Fuge including its dimensions.

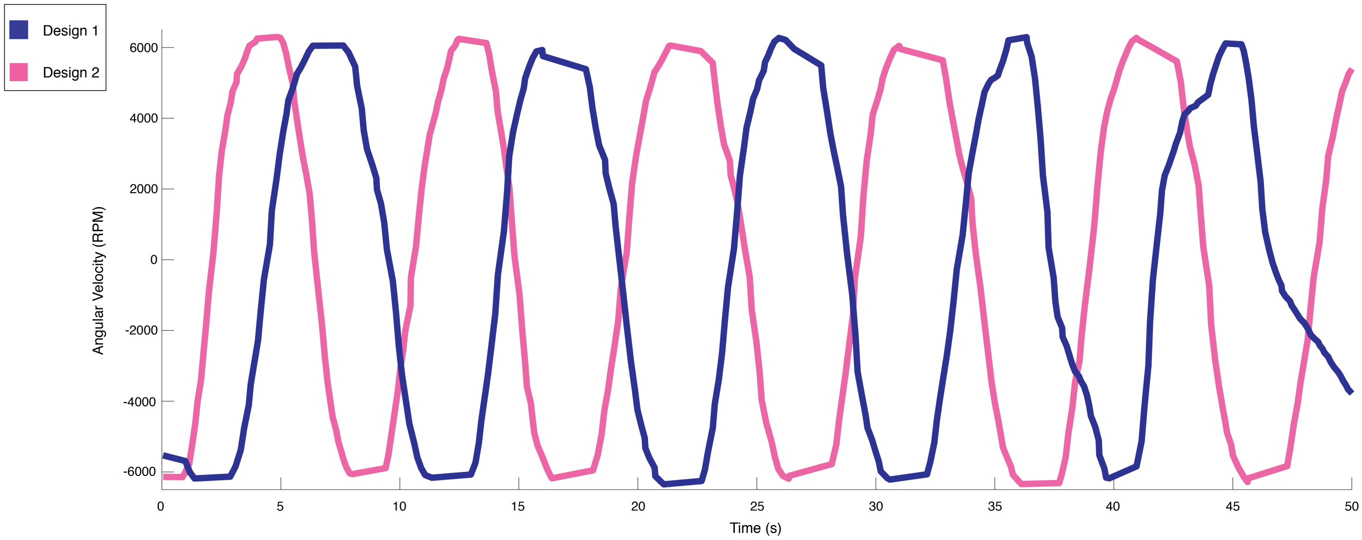


FIG. S4. **RPM values of 3D-Fuge.** Shows the RPM of both designs over a cycle of 5 runs. Both designs have similar peak RPM at around 6000; however, they have slightly different periods of revolution. The data shows reproducible rpm cycles with time.

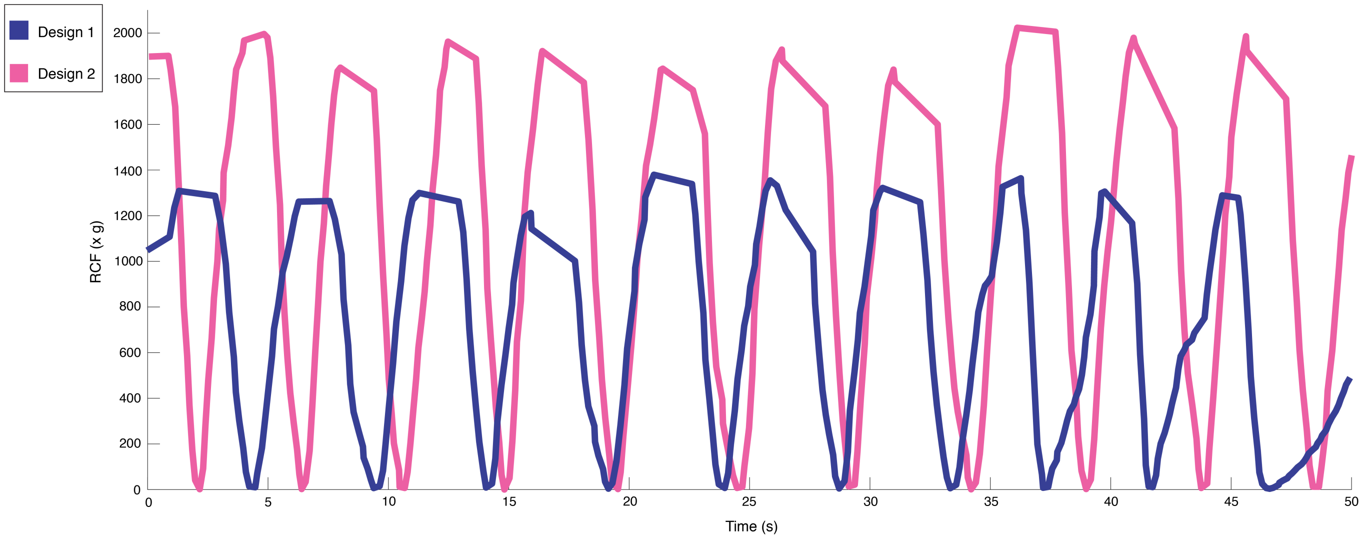


FIG. S5. **RCF values of 3D-Fuge.** Shows the RCF of both designs over a cycle of 5 runs. Although both the designs have the same rpm values, Design 1 has a smaller g-force due to its smaller radius.
